# New ibuprofen derivatives with thiazolidine-4-one scaffold with improved pharmaco-toxicological profile

**DOI:** 10.1186/s40360-021-00475-0

**Published:** 2021-02-04

**Authors:** Ioana-Mirela Vasincu, Maria Apotrosoaei, Sandra Constantin, Maria Butnaru, Liliana Vereștiuc, Cătălina-Elena Lupușoru, Frederic Buron, Sylvain Routier, Dan Lupașcu, Roxana-Georgiana Taușer, Lenuța Profire

**Affiliations:** 1 Pharmaceutical Chemistry Department, Faculty of Pharmacy, University of Medicine and Pharmacy “Grigore T. Popa” of Iasi, Iași, Romania; 2 Biomedical Sciences Department, Faculty of Medical Bioengineering, University of Medicine and Pharmacy “Grigore T. Popa” of Iasi, Iași, Romania; 3 Pharmacology Department, Faculty of Medicine, University of Medicine and Pharmacy “Grigore T. Popa” of Iasi, Iași, Romania; 4 Institute of Organic and Analytical Chemistry, Université d’Orléans - Pôle de chimie, Orléans, France

**Keywords:** Ibuprofen, Thiazolidine-4-one, Toxicity degree, Anti-inflammatory and analgesic effects

## Abstract

**Background:**

Aryl-propionic acid derivatives with ibuprofen as representative drug are very important for therapy, being recommended especially for anti-inflammatory and analgesic effects. On other hand 1,3-thiazolidine-4-one scaffold is an important heterocycle, which is associated with different biological effects such as anti-inflammatory and analgesic, antioxidant, antiviral, antiproliferative, antimicrobial etc. The present study aimed to evaluated the toxicity degree and the anti-inflammatory and analgesic effects of new 1,3-thiazolidine-4-one derivatives of ibuprofen.

**Methods:**

For evaluation the toxicity degree, cell viability assay using MTT method and acute toxicity assay on rats were applied. The carrageenan-induced paw-edema in rat was used for evaluation of the anti-inflammatory effect while for analgesic effect the tail-flick test, as thermal nociception in rats and the writhing assay, as visceral pain in mice, were used.

**Results:**

The toxicological screening, in terms of cytotoxicity and toxicity degree on mice, revealed that the ibuprofen derivatives (**4a-n**) are non-cytotoxic at 2 μg/ml. In addition, ibuprofen derivatives reduced carrageenan-induced paw edema in rats, for most of them the maximum effect was recorded at 4 h after administration which means they have medium action latency, similar to that of ibuprofen. Moreover, for compound **4d** the effect was higher than that of ibuprofen, even after 24 h of administration. The analgesic effect evaluation highlighted that **4 h** showed increased pain inhibition in reference to ibuprofen in thermal (tail-flick assay) and visceral (writhing assay) nociception models.

**Conclusions:**

The study revealed for ibuprofen derivatives, noted as **4 m, 4 k, 4e, 4d**, a good anti-inflammatory and analgesic effect and also a safer profile compared with ibuprofen. These findings could suggest the promising potential use of them in the treatment of inflammatory pain conditions.

## Background

Non-steroidal anti-inflammatory drugs (NSAIDs) in which aryl-propionic acid derivatives (ibuprofen, fenoprofen, ketoprofen, naproxen, etc.) have an important place, being among the most widely used pain drugs to treat pain and inflammation associated with rheumatic diseases but also the other types of pain such as renal colic, biliary colic, headache, dysmenorrhea, etc. [1].

Moreover, recent researches bring solid arguments regarding the beneficial effects of NSAIDs in neurodegenerative and cancer diseases, in which, inflammation and over-production of pro-inflammatory cytokines, as well as the oxidative stress, play an important role [2].

The epidemiological studies showed that more than 35 million people take daily NSAIDs and 40% of them are aged over 60 years. It also should be noted that annual sales of NSAIDs reach huge amounts that exceed $6 billion and for Europe NSAIDs prescriptions are more than 7.5% of all prescriptions issued in a year [1, 3].

On the other hand thiazolidine-4-one scaffold is one of the heterocycles that is widespread in the organic chemistry, being responsible for many biological effects [4] such as: anti-inflammatory and analgesic, antifungal and antimicrobial, anti-mycobacterial, antioxidant, antiviral and anti-HIV, anticonvulsant, hypoglycemic and antitumor effects [5].

Current researches aim the design and synthesis of hybrid molecules containing two or more pharmacophore scaffolds in order to improve the properties of the classical drugs or to induce new biological effects, providing in the same time a safer profile [6].

The aim of this work was to investigate the biological effects of the new derivatives of ibuprofen with thiazolidine-4-one scaffold synthesized by our research group [7], targeting the toxicity degree and the anti-inflammatory and analgesic effects.

## Methods

### Chemicals and reagents

**Fig. 1:**
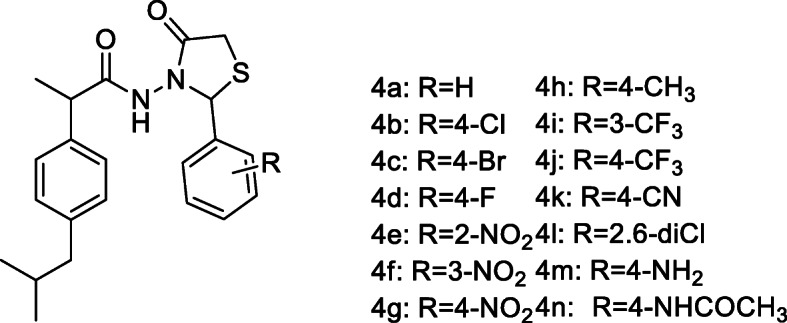
The structure of ibuprofen derivatives with thiazolidine-4-one scaffold (**4a-n**)

Dulbecco’s Modified Eagle Medium (DMEM) with 4500 mg/ml glucose, 110 mg/l sodium pyruvate and 0.584 mg/l L-glutamine; Bovine Fetal Serum (BFS); Penicillin/ streptomycin/neomycin (P/S/N) solution with 5000 units penicillin, 5 mg streptomycin and 10 mg neomycin/ml; phosphate buffered saline; 3-(4,5-dimethyl-2-thiazolyl)-2,5-diphenyl-2H-tetrazolium bromide (MTT), ibuprofen, dimethylsulfoxide (DMSO), acetic acid, tween 80, k-carrageenan. All materials and reagents were purchased from Sigma-Aldrich. The thiazolidin-4-one derivatives of ibuprofen (**4a-n**, Fig. 1) were previously synthesized and characterized by our research group [7].

### Animals

Swiss albino mice and Wistar rats provided from the Biobase of “Grigore T. Popa” University of Medicine and Pharmacy from Iasi, were used. The animals were housed in polyethylene cages with access to water and food ad libitum for 7 days before starting the experiments. The environmental conditions during the study were maintained relatively constant at a 23 ± 2 °C temperature, 40–60% relative humidity and a 12 h light/dark cycle. The food and the water were withdrawn 18 h before starting the experiments. The animals (mice and rats respectively) were randomly divided into several groups (*n* = 8), depending of the method applied. The inclusion criteria used in the design of the experiment refer to the weight of animal (20–30 g for mice, 150–200 g for rats) and healthy state and the exclusion criteria applied were any pathological conditions observed to the animals. All the experiments were designed to cause minimum harm to animals. At the end of the experiments, the animals were anesthetized with ethyl ether and then the cervical dislocation procedure was used to euthanasia the animals. Prior to the disposal, the animal death was confirmed by observing the movement, heartbeat, respiration and eye reflex. All procedures were strictly conducted by the expert personnel and were in agreement with the guideline of laboratory animal studies.

### Ethics approval and consent to participate

The study was conducted in agreement with actual deontology and ethics guidelines about laboratory animal studies (Law no. 206/27 May 2004, EU/2010/63 - CE86/609/EEC) and was approved by Research Ethics Committee of “Grigore T. Popa” University of Medicine and Pharmacy from Iasi (resolution no. 292).

**Cell viability assay** using the MTT method is based on the capacity of cell to reduce the slightly yellow tetrazolium salts to intense purple formazan by intracellular reduction system mostly located in the mitochondria. The amount of formazan, which is correlated with number of viable cells, is spectrophotometrically measured at 570 nm [8]. Primary cells, mesenchymal type with stem cell potential, isolated by collagenization from adipose tissue, were used. The cell line was maintained in DMEM supplemented with BFS (10%) and P/S/N in a humidified incubator with 5% CO_2_ at 37 °C. The cells were seeded in 24-well plates (10^4^ cells/ml) and treated after 24 h with different concentrations of ibuprofen derivatives (**4a-n**) (50 μg/ml, 10 μg/ml, 2 μg/ml) for 24 h, 48 h and 72 h. DMSO was used as solvent and a negative control (blank, DMSO 0.2%) and a positive control (DMSO 5%) were used in similar conditions. The culture media was removed and MTT (500 μl) was next added to each well and the cells were further incubated for 3 h at 37 °C. The medium was then removed and isopropanol was added. After 15 min from each well were transferred 100 μl in a 96-well plate and the absorbance was recorded at 570 nm with a microplate reader [9, 10]. The cell viability rate was calculated using the following formula [9]:

$$ \mathrm{Cell}\ \mathrm{viability}\ \left(\%\right)=\left({\mathrm{A}}_{\mathrm{s}}/{\mathrm{A}}_{\mathrm{c}}\right)\times 100 $$

in which,

A_s_ = the absorbance of culture cells incubated with the sample (ibuprofen derivatives);

A_c_ = the absorbance of culture cells incubated with DMSO (0.2%).

The experiments were performed in triplicate and the results are presented as mean ± standard deviation (SD).

**Acute toxicity assay** was performed on mice and the tested compounds (**4a-n**) were suspended in tween 80 and administrated orally, in volume of 0.1 ml/10 g animal. The different doses (1000–3000 mg/kg body weight) of the tested compounds were used [11] and the survival rate was noted at the different timelines: 24, 48, 72 h and 7 and 14 days. The LD_50_ was calculated on base of Kärber arithmetic method [12] using the following formula:

$$ {\mathrm{LD}}_{50}={\mathrm{LD}}_{100}-\left(\sum \left(\mathrm{a}+\mathrm{b}\right)/\mathrm{n}\right), $$

in which:

a = the difference between two succesive doses of tested compounds;

b = the average number of dead animals in two successive doses;

*n =* the number of animals for each group;

LD_100_ = the lethal dose causing the 100% death of all test animals.

***Carrageenan-induced paw edema assay*** was used to evaluate the anti-inflammatory effect, according to the protocols describes in the literature [13, 14] with slightly modifications. The edema was induced by intra-plantar administration of 0.2 ml of 1% suspension of k-carrageenan in physiological saline into the left hind paw of rat and the volume of paw was measured using the digital pletismometer LE. After induction of edema, the ibuprofen derivatives (**4a-n**) were administered orally in a daily dose representing 1/20 of LD_50_ as suspension in tween 80 (0.5 mL/100 g b.w.). Ibuprofen as reference drug and tween 80 (0.5 mL/100 g b.w.) as control, were used in similar conditions (Table 1).

**Table 1:** The doses (mg/kg b.w.) of ibuprofen derivatives (**4a-n**) used for anti-inflammatory and analgesic assays

Group	Compound	Dose (mg/kg b.w.)	Group	Compound	Dose (mg/kg b.w.)
1	**4a**	81.25	9	**4i**	86.00
2	**4b**	81.25	10	**4j**	92.00
3	**4c**	86.40	11	**4 k**	78.25
4	**4d**	81.70	12	**4 l**	79.50
5	**4e**	91.00	13	**4 m**	82.50
6	**4f**	89.50	14	**4n**	85.00
7	**4 g**	91.00	15	**Ibuprofen**	68.75
8	**4 h**	89.50	16	**Tween 80**	0.5 ml/100 g

The volume of the left hind paw was measured at different timelines (2, 4, 6 and 24 h). The edema inhibition (%) was calculated, using the following formula:

$$ \mathrm{Edema}\ \mathrm{inhibition}\ \left(\%\right)=\left(\Delta {\mathrm{V}}_{\mathrm{c}}-\Delta {\mathrm{V}}_{\mathrm{t}}\right)\ \mathrm{x}\ 100/\Delta {\mathrm{V}}_{\mathrm{c}} $$

in which:

ΔV_c_ = the rat paw’s volume recorded for control group;

ΔV_t_ = the rat paw’s volume recorded for groups treated with ibuprofen derivatives.

#### Analgesic effect

*Tail-flick assay*, used as model of thermal nociception is based on measuring the sensibility of rats when a thermal stimulus is applied on tail. According to the experimental protocol, the animals were initially tested by applying a radiant beam at the distal part of the tail, measuring the latency at time 0 (T_0_) [15, 16]. The response to pain was quantified using a Tail-flick algesimeter (Harvard Apparatus, United States of America). The ibuprofen derivatives (**4a-n**) were administered by oral gavage in a daily dose representing 1/20 of LD_50_, as suspension in tween 80 (0.5 mL/100 g b.w.) (Table 1). Ibuprofen, as reference drug, was used in similar conditions. In order to assess the analgesic effect, the initial response to pain and at 4 h after administration of ibuprofen derivatives (**4a-n**) it was determined. The maximum allowed time (cut-off time), for not causing tissue lesions was established to be 10 s (T_m_).

The pain inhibition (%) was calculated for each tested compound, using the following formula:

$$ \mathrm{Pain}\ \mathrm{inhibition}\ \left(\%\right)=\left({\mathrm{T}}_{\mathrm{t}}-{\mathrm{T}}_0\right)/\left({\mathrm{T}}_{\mathrm{m}}-{\mathrm{T}}_0\right)\ \mathrm{x}\ 100 $$

in which:

T_t_ = the nociceptive response measured at 4 h after of ibuprofen derivatives administration;

T_0_ = the nociceptive response measured before any treatment (initially);

T_m_ = the maximum allowed time (cut-off time).

The presence of analgesic effect (anti-nociceptive potential) is highlighted by an increased latency response after administration of the ibuprofen derivatives in reference with the initially value.

*Writhing assay* is a model of visceral pain, induced to mice by intraperitoneal administration of acetic acid. It is characterized through abdominal contractions, body movements (especially of the posterior members), writhing of the dorsal-abdominal muscles with reduced locomotor activity. The applied experimental protocol was in agreement with the literature data with slightly modifications [17, 18]. The ibuprofen derivatives (**4a-n**) were administered by oral gavage in a daily dose representing 1/20 of LD_50_ as suspension in tween 80 (0.1 mL/10 g b.w.) (Table 1). Ibuprofen, as reference drug and tween 80 (0.1 ml/10 g b.w.), as control, were used in similar conditions. One hour after administration of tested compounds, the acetic acid (0.6% water solution), as irritating agent, in volume of 0.1 ml/10 g b.w. was intraperitoneally injected. After other 5 min the number of writhings for each mouse was noted, every 5 min, during 30 min. The analgesic effect, expressed as inhibition (%) of writhings was calculated for each tested compounds, using the following formula [14]:

$$ \mathrm{Inhibition}\ \left(\%\right)=\left({\mathrm{N}}_{\mathrm{c}}-{\mathrm{N}}_{\mathrm{t}}\right)\ \mathrm{x}\ 100/{\mathrm{N}}_{\mathrm{c}} $$

in which:

N_c_ = the writhings number recorded for mice from control group.

N_t_ = the writhings number recorded for mice of group treated with ibuprofen derivatives.

It is estimated that the analgesic activity is higher if the writhings number is decreased in comparison with control group.

### Statistical analysis

Data are presented as mean ± standard deviation. The one-way analysis of variance (ANOVA) and Tukey post hoc test were used to determine whether there are any statistically significant differences between tested compounds and control. The *p* value < 0.05 was considered to be statistically significant.

## Results

### Cell viability assay

The cell viability values recorded for tested compounds (**4a-n**) at different concentrations (50 μg/ml, 10 μg/ml and 2 μg/ml) and at different timelines (24, 48 and 72 h) are presented at Tables 2, 3 and 4.

At 50 μg/ml was evidenced decreasing of cell viability with exposure time and the resulting percentages varied between 1.40 ± 0.26% to 64.25 ± 1.06% (24 h), from 1.14 ± 0.24% to 56.18 ± 0.88% (48 h) and from 0.34 ± 0.09% to 53.12 ± 1.65% (72 h). At this concentration, **4 m** (*R =* 4-NH_2_) and **4n** (*R =* 4-NHCOCH_3_), were the least toxic (Table 2).

**Table 2:** Cell viability (%) of ibuprofen derivatives (**4a-n**) at 50 μg/ml

Comp.	24 h	48 h	72 h	Comp.	24 h	48 h	72 h
**4a**	2.41 ± 0.40	1.51 ± 0.18	0.45 ± 0.07	**4 h**	5.77 ± 0.25	3.53 ± 0.20	3.01 ± 0.32
**4b**	2.06 ± 0.16	1.64 ± 0.33	0.34 ± 0.09	**4i**	1.40 ± 0.26	1.87 ± 0.22	1.28 ± 0.21
**4c**	5.43 ± 0.29	2.78 ± 0.12	3.42 ± 0.23	**4j**	4.43 ± 0.14	2.96 ± 0.12	2.65 ± 0.08
**4d**	5.85 ± 0.38	3.32 ± 0.12	3.63 ± 0.08	**4 k**	2.48 ± 0.67	1.17 ± 0.11	1.51 ± 0.38
**4e**	6.27 ± 0.35	2.58 ± 0.11	1.14 ± 0.08	**4 l**	15.56 ± 1.56	3.71 ± 0.10	3.27 ± 0.35
**4f**	2.02 ± 0.35	1.14 ± 0.24	1.09 ± 0.19	**4 m**	59.89 ± 1.31	56.18 ± 0.88	53.12 ± 1.65
**4 g**	7.36 ± 0.52	3.23 ± 0.18	3.01 ± 0.53	**4n**	64.25 ± 1.06	54.87 ± 0.89	52.37 ± 1.84
**Ibuprofen**	91.78 ± 2.41	90.46 ± 1.11	87.70 ± 0.29	**DMSO 5%**	36.43 ± 1.45	14.40 ± 1.00	6.95 ± 0.38

An improving of cell viability was observed at 10 μg/ml, the values recorded ranging from 51.95 ± 1.42% to 97.51 ± 2.01% (24 h), from 41.04 ± 0.12 to 90.51 ± 0.66% (48 h) and from 41.03 ± 0.62% to 77.68 ± 1.59% (72 h) respectively. The less toxic derivatives were **4f** (*R =* 3-NO_2_, 97.51 ± 2.01%), **4 k** (*R =* 4-CN, 92.29 ± 0.33%) and **4n** (*R =* 4-NHCOCH_3_, 95.25 ± 1.74%), the cell viability values being slightly increased (**4f**, **4n**) or comparable (**4 k**) to ibuprofen (92.83 ± 2.24%) (Table 3).

**Table 3:** Cell viability (%) of ibuprofen derivatives (**4a-n**) at 10 μg/ml

Comp.	24 h	48 h	72 h	Comp.	24 h	48 h	72 h
**4a**	61.36 ± 1.17	59.69 ± 1.23	57.62 ± 1.26	**4 h**	80.38 ± 1.39	74.95 ± 1.86	64.76 ± 1.42
**4b**	56.38 ± 1.50	52.01 ± 0.73	38.76 ± 0.76	**4i**	89.03 ± 0.66	78.43 ± 1.17	77.16 ± 1.47
**4c**	82.20 ± 1.24	79.39 ± 1.01	69.86 ± 1.86	**4j**	51.95 ± 1.42	41.04 ± 0.12	41.03 ± 0.62
**4d**	80.94 ± 0.88	80.74 ± 1.39	76.88 ± 1.61	**4 k**	92.29 ± 0.33	76.04 ± 0.95	70.97 ± 1.58
**4e**	84.79 ± 2.01	78.33 ± 0.95	71.19 ± 1.68	**4 l**	68.71 ± 1.49	62.60 ± 1.14	60.24 ± 1.77
**4f**	97.51 ± 2.01	90.51 ± 0.66	76.71 ± 1.68	**4 m**	76.89 ± 2.01	75.77 ± 1.52	61.19 ± 1.05
**4 g**	85.95 ± 0.89	78.06 ± 1.77	71.47 ± 1.93	**4n**	95.25 ± 1.74	81.10 ± 0.63	77.68 ± 1.59
**Ibuprofen**	92.83 ± 2.24	91.65 ± 1.68	90.46 ± 1.12	**DMSO 5%**	36.43 ± 1.45	14.40 ± 1.00	6.95 ± 0.38

For all compounds (**4a-n**) the cell viability values were higher than 70% at 2 μg/ml so they are considered non-cytotoxic [19] at this concentration (Table 4). The values of cell viability were ranged from 85.06 ± 2.01% to 99.64 ± 1.89% (24 h), from 77.07 ± 2.06% to 99.61 ± 1.53% (48 h) while the values were placed in 73.84–95.02% interval at 72 h. After 72 h the less toxic were **4 m** (*R =* 4-NH_2_, 95.02 ± 1.46% **4f** (*R =* 3-NO_2_, 94.44 ± 0.10%), **7c** (*R =* 4-Br, 94.39 ± 1.65%), **4n** (*R =* 4-NHCOCH_3_, 93.91 ± 1.96%).

**Table 4:** Cell viability (%) of ibuprofen derivatives (**4a-n**) at 2 μg/ml

Comp.	24 h	48 h	72 h	Comp.	24 h	48 h	72 h
**4a**	88.47 ± 1.70	84.65 ± 1.17	78.83 ± 1.29	**4 h**	98.52 ± 0.88	97.98 ± 2.16	92.30 ± 1.19
**4b**	85.06 ± 2.01	77.07 ± 2.06	73.84 ± 1.19	**4i**	90.80 ± 1.89	84.62 ± 1.24	83.89 ± 2.08
**4c**	99.56 ± 1.46	94.88 ± 0.71	94.39 ± 1.65	**4j**	91.71 ± 1.95	91.07 ± 1.19	89.45 ± 1.01
**4d**	96.63 ± 1.77	90.17 ± 1.01	84.98 ± 0.65	**4 k**	89.70 ± 2.17	89.56 ± 0.15	87.85 ± 1.26
**4e**	89.24 ± 1.24	88.86 ± 1.53	75.31 ± 1.89	**4 l**	93.85 ± 1.14	92.14 ± 2.06	84.46 ± 1.59
**4f**	99.48 ± 0.63	98.46 ± 1.55	94.44 ± 0.10	**4 m**	99.64 ± 1.89	99.61 ± 1.53	95.02 ± 1.46
**4 g**	97.46 ± 1.52	95.75 ± 1.95	85.62 ± 0.37	**4n**	96.01 ± 1.14	95.00 ± 1.59	93.91 ± 1.96
**Ibuprofen**	98.48 ± 2.44	97.39 ± 1.30	92.64 ± 1.96	**DMSO 5%**	36.43 ± 1.45	14.40 ± 1.00	6.95 ± 0.38

### Acute toxicity assay

Referring to the in vivo toxicity degree the data revealed that all tested compounds have showed to be less toxic compared with ibuprofen (LD_50_ = 1375 mg/kg b.w.) having the LD_50_ values ranged between 1565 mg/kg b.w. and 1840 mg/kg b.w. (Table 5). The less toxic derivatives were **4j** (*R =* 4-CF_3_, LD_50_ = 1840 mg / kg b.w.), **4e** (*R =* 2-NO_2_, LD50 = 1820 mg/kg b.w.) and **4 g** (*R =* 4-NO_2_, LD50 = 1820 mg/kg b.w.) that proved to be about 1.3 times less toxic than ibuprofen.

**Table 5:** The values of LD_50_ recorded for ibuprofen derivatives (**4a-n**)

Comp.	LD_**50**_ (mg/kg b.w.)	Comp.	LD_**50**_ (mg/kg b.w.)
**4a**	1625	**4 h**	1790
**4b**	1625	**4i**	1720
**4c**	1728	**4j**	1840
**4d**	1634	**4 k**	1565
**4e**	1820	**4 l**	1590
**4f**	1790	**4 m**	1650
**4 g**	1820	**4n**	1700
**Ibuprofen**	1375		

### Carrageenan-induced paw edema assay

**Fig. 2:**
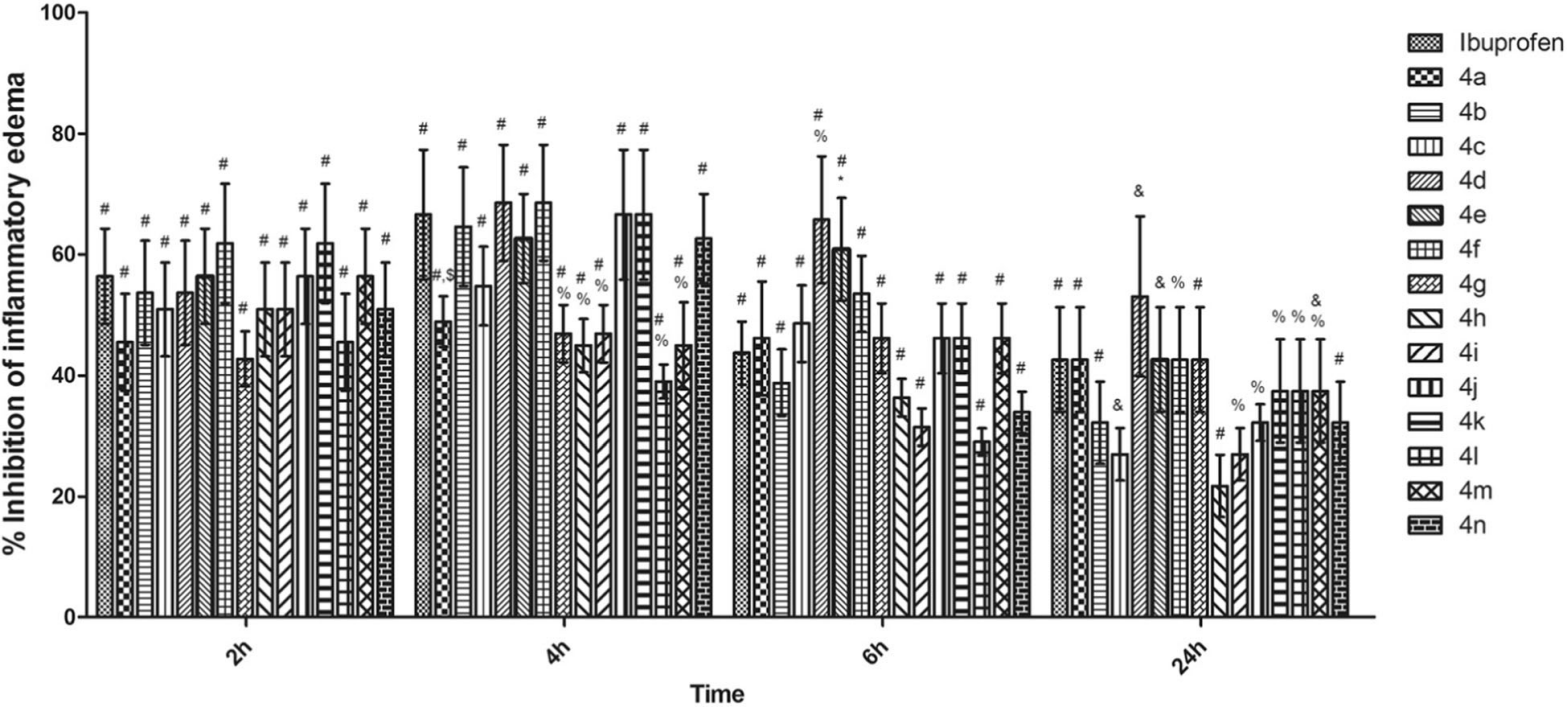
The edema inhibition (%) recorded for ibuprofen derivatives (**4a-n**) in comparison with ibuprofen at different timelines (the data represent the mean of 8 values ± standard deviation). One-way analysis of variance (ANOVA) followed by Tukey post hoc test was performed. & - *p* < 0.01 vs. vehicle, # - *p* < 0.001 vs. vehicle, * - *p* < 0.05 vs. ibuprofen, $ - *p <* 0.01 vs. ibuprofen, % - *p <* 0.001 vs. ibuprofen

The new synthesized ibuprofen derivatives (**4a-n**) were tested at a dose of 1/20 LD_50_ and the results, expressed as edema inhibition (%) are show in Fig. 2. It can be noticed that at 2 h after administration, the edema inhibition (%) varied between 42.72 ± 4.55% and 61.81 ± 9.87%, for most part of the tested compounds the effect being comparable to the ibuprofen one (56.36 ± 7.87%). At this time the most active compounds were **4f** (*R =* 3-NO_2_) and **4 k** (*R =* 4-CN), for which the value of edema inhibition (%) was 61.81 ± 9.87%, slightly higher than ibuprofen. A noticeable activity, similar to that of ibuprofen, showed also **4e** (*R =* 2-NO_2_), **4j** (*R =* 4-CF_3_) and **4 m** (*R =* 4-NHCOCH_3_), for which the value of edema inhibition was 56.36 ± 7.87%. The effect remained in quite similar range at 4 h after administration, the edema inhibition (%) ranging between 39.01 ± 2.81% and 68.52 ± 9.57%. At this timeline an appreciable effect was noted in case of **4d** (*R =* 4-F) and **4f** (*R =* 3-NO_2_), for which the edema inhibition (%) was 68.52 ± 9.57%. A similar effect was noted for **4j** (*R =* 4-CF_3_) and **4 k** (*R =* 4-CN), for which the inhibition edema (%) was 66.55 ± 10.72%. In similar condition the edema inhibition recorded for ibuprofen was 66.55 ± 10.72%.

At 6 h after administration the most active compounds were **4d** (*R =* 4-F) and **4e** (*R =* 2-NO_2_) for which the inhibition percentages of paw edema were 65.71 ± 10.49% and 60.81 ± 8.49%, being higher than ibuprofen value (43.67 ± 5.20%). The analysis of the data recorded at 24 h after administration, revealed a long lasting anti-inflammatory effect for tested derivatives, even slightly higher than ibuprofen for some of them. The most active compound proved to be **4d** (*R =* 4-F), with an edema inhibition value of 53.04 ± 13.17%.

### Analgesic effect

#### Tail-flick assay

**Fig. 3:**
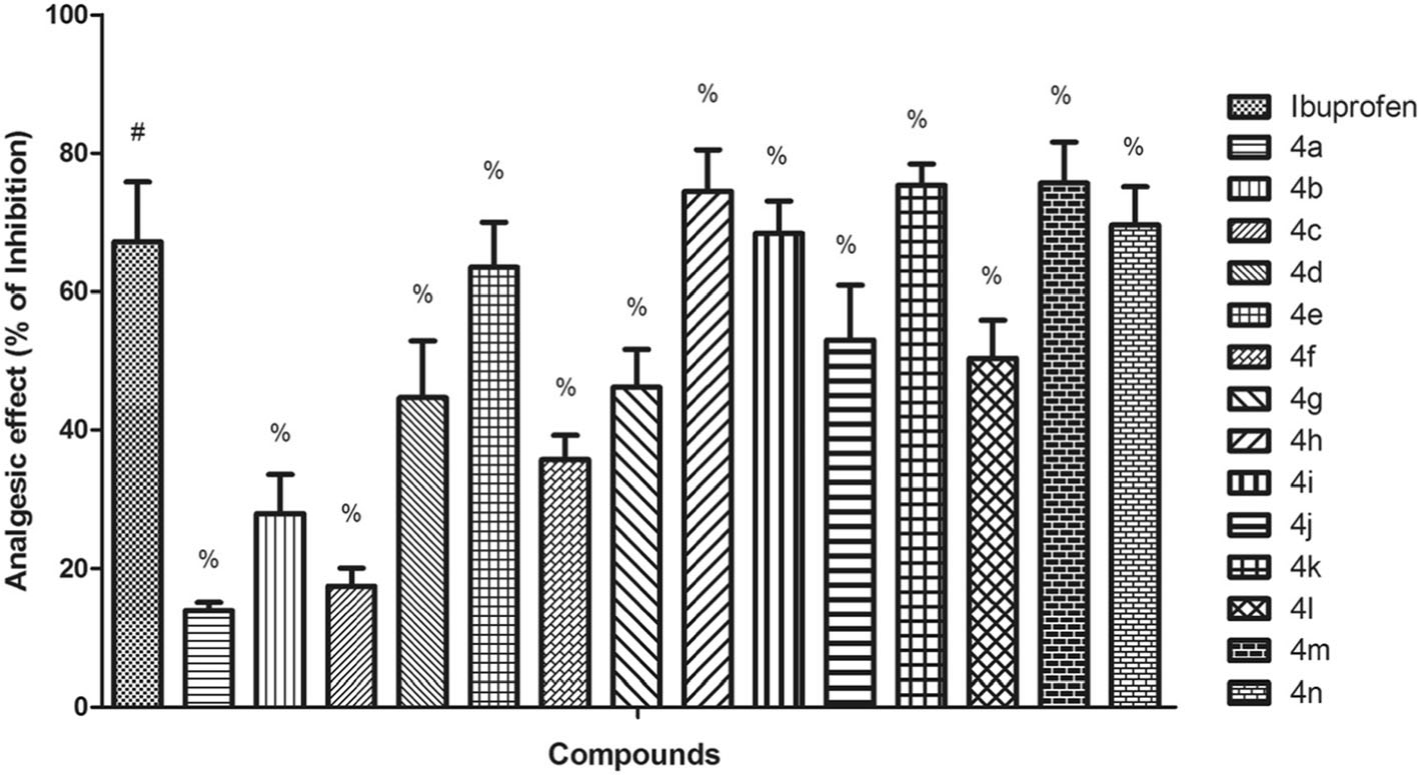
The pain inhibition (%) recorded for ibuprofen derivatives (4**a-n**) in comparison with ibuprofen at different timelines (the data represent the mean of 8 values ± standard deviation). One-way analysis of variance (ANOVA) followed by Tukey post hoc test was performed. # - *p <* 0.001 vs. vehicle, % - *p <* 0.001 vs. ibuprofen

Based on the value of reaction time of animals from the control group and the value of the maximum set time (10 s), for each group treated with ibuprofen derivatives (**4a-n**), the maximum effect, expressed as pain inhibition (%) was calculated (Fig. 3). The pain inhibition (%) recorded for ibuprofen derivatives ranged between 13.86 ± 1.19% and 75.67 ± 5.94%, while for ibuprofen the recorded value was 67.15 ± 8.66. The most active compounds proved to be **4 m** (*R =* 4-NH_2_,75.67 ± 5.94%), **4 k** (*R =* 4-CN, 75.36 ± 3.08%) and **4 h** (*R =* 4-CH_3_, 74.45 ± 6.06%) for which the pain inhibition effect was higher than of that of ibuprofen.

The compounds **4n** (*R =* 4-NHCOCH_3_, 69.59 ± 5.56%), **4i** (*R =* 3-CF_3_, 68.37 ± 4.70%) and **4e** (*R =* 2-NO_2_, 63.50 ± 6.49%) also showed an appreciable pain inhibitory effect, comparable with that of ibuprofen.

#### Writhing assay

**Fig. 4:**
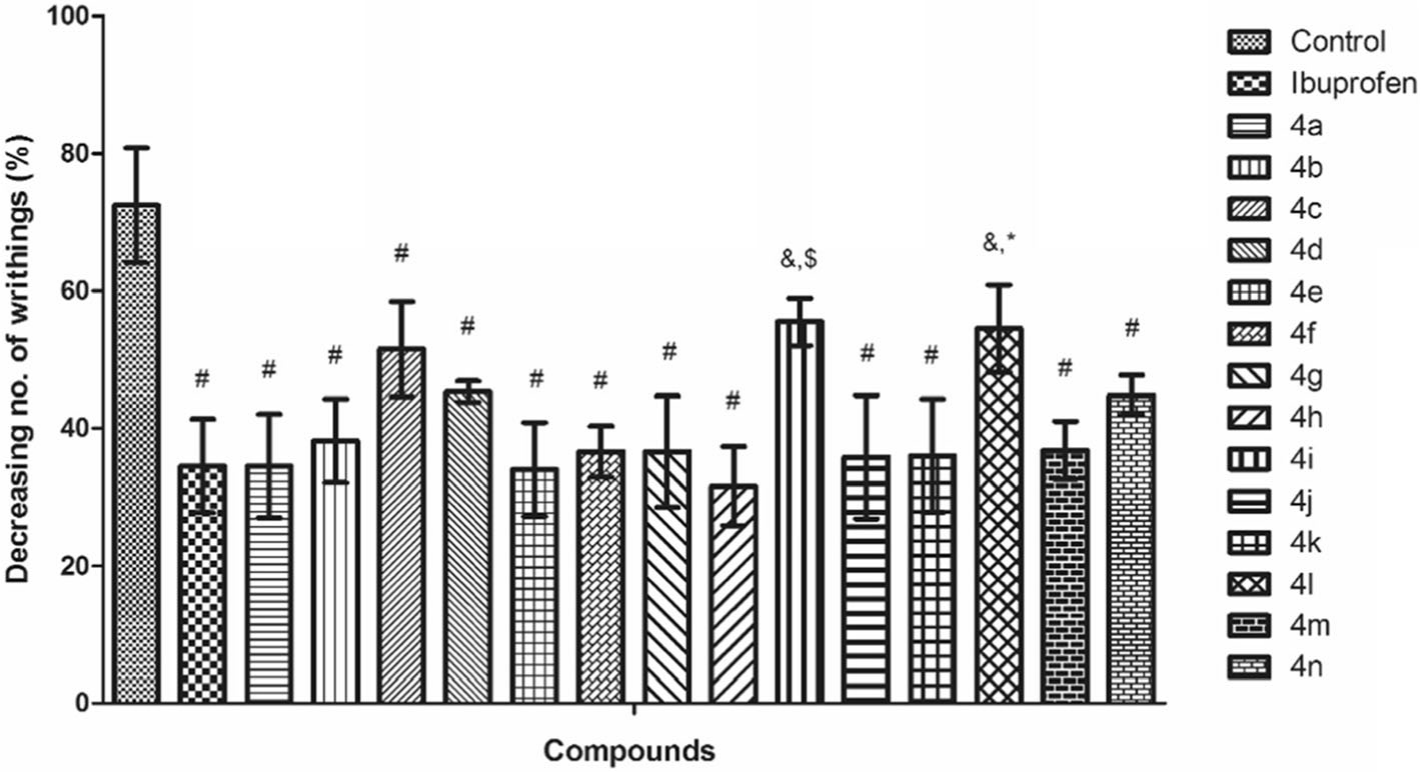
The inhibition of writhings (%) recorded for ibuprofen derivatives (**4a-n**) in comparison with ibuprofen (the data represent the mean of 8 values ± standard deviation). One-way analysis of variance (ANOVA) followed by Tukey post hoc test was performed. & - *p <* 0.01 vs. vehicle, # - *p <* 0.001 vs. vehicle, * - *p <* 0.05 vs. ibuprofen, $ - *p <* 0.01 vs. ibuprofen

From the analysis of the obtained results (Fig. 4) it is noticed a decrease of the writhings number in case of groups treated with ibuprofen derivatives (**4a-n**) in comparison to the control group, which means they could be appreciated having good analgesic effects.

The analgesic effect, expressed as inhibition of writhing number, was 52.37 ± 10.33% for ibuprofen while for the ibuprofen derivatives the recorded values varied between 23.38 ± 1.45% and 56.37 ± 10.30%. The most intense peripheral analgesic effect, higher than that of ibuprofen was recorded for **4 h** (*R =* 4-CH_3_, 56.37 ± 10.30) and **4e** (*R =* 2-NO_2_, 53.06 ± 10.63). Appreciable effects, comparable to that of ibuprofen, were showed also by **4a** (*R =* H, 52.37 ± 11.39), **4j** (*R =* 4-CF_3_, 50.58 ± 12.69), **4 k** (*R =* 4-CN, 50.30 ± 11.52), **4f** (*R =* 3-NO_2_, 49.47 ± 5.02), **4 g** (*R =* 4-NO_2_, 49.47 ± 10.92), **4 m** (*R =* 4-NH_2_, 49.19 ± 5.62) and **4b** (*R =* 4-Cl, 47.31 ± 7.54).

## Discussion

Ibuprofen is a widely used nonsteroidal anti-inflammatory drug which belongs to aryl-propionic acid derivatives. It acts mainly as nonselective inhibitor of both cyclooxygenase (COX) enzymes (COX-1 and COX-2). Its free carboxyl group allows a variety of structural modification and is also responsible for a part of side effects that could appear at gastric, renal or liver level. It was noted that modifying the carboxyl group could lead to compounds that become more selective for COX-2 isoform. Some pharmacophores such as two aromatic rings seem to be also responsible for COX-2 selectivity by fitting in the enzyme structure [20]. Starting from ibuprofen, new thiazolidine-4-one derivatives have been synthesized by our research group [7], as candidates to alleviate the pain or inflammation associated with various pathological conditions. To prove the therapeutic potential of the synthesized derivatives a pharmaco-toxicological screening that has included in vitro and in vivo assays, was performed.

The MTT cell viability assay is an in vitro colorimetric assay, which determines the mitochondria activity, hence providing information on cellular energy metabolism [9, 10]. It was observed that the values of cell viability recorded for tested compounds (**4a-n**) at 50 μg/ml decrease in time, a good cell viability being recorded for **4 m** (*R =* 4-NH_2_) and **4n** (*R =* 4-NHCOCH_3_) at all timelines (24 h, 48 h, 72 h). An improving of cell viability was observed at 10 μg/ml, for some derivatives (**4f**, *R =* 3-NO_2_; **4 k**, *R =* 4-CN and **4n**, *R =* 4-NHCOCH_3_) the value recorded being comparable with of ibuprofen one, especially at 24 h. At 2 μg/ml, all tested compounds (**4a-n**) are considered non-cytotoxic because the values of cell viability are higher than 70% and comparable with ibuprofen at all timelines. The leader compound in view of citotoxicity seems to be **4n**, for which the values of cell viability recorded for all tested concentrations (50 μg/ml, 10 μg/ml and 2 μg/ml) support its non-citotoxicity. These results support the favorable influence of NHCOCH_3_ substituent on aromatic ring in view of decreasing of the cytotoxicity degree.

The acute toxicity, expressed by median lethal dose 50 (LD_50_), represents the base for toxicological classification of substances [21, 22]. It is knows that toxicity is responsible for many side effects, which could appear either immediately or after a time, following administration of a single dose or multiple doses of the substance in 24 h. All tested ibuprofen derivatives (**4a-n**) are slightly toxic, the values of LD_50_ being higher than of ibuprofen one. So, it could be appreciated that chemical modulation of ibuprofen using thiazolidine-4-one scaffold leads to decreasing of the toxicity. In addition, the substitution of aromatic ring of thiazolidine-4-one scaffold seems to have favorable influence for reducing the toxicity degree.

Carrageenan-induced paw edema is a widely used experimental acute inflammation model to assess the anti-inflammatory action of compounds designed as potential anti-inflammatory agents [23–26]. This model involves many mediators such as prostaglandins, cytokines, histamine and bradykinin that stimulate the inflammatory process [27–29]. Our study demonstrated that all tested ibuprofen derivatives reduced paw edema, for the most part of them the effect being comparable to that of ibuprofen, used as reference drug. For the most of them (**4a-g**, **4j**, **4 k**, **4n**) the maximum effect was recorded after 4 h of administration, similar to ibuprofen, which means they are considered compounds with medium action latency. For **4 h** (*R =* 4-CH_3_), **4i** (*R =* 3-CF_3_), **4 l** (*R =* 2,6-diCl) and **4 m** (*R =* 4-NH_2_) the maximum effect was recorded after 2 h of administration, which means they have short action latency. The most proper compounds proved to be **4d** (*R =* 4-F), **4a** (*R =* −H), **4e** (*R =* 2-NO_2_), **4 g** (*R =* 4-NO_2_) and **4f** (*R =* 3-NO_2_), for which a long-term anti-inflammatory effect was recorded. In addition, the effect of these compounds was higher or comparable to that of ibuprofen, used as reference drug, at all studied time intervals (2, 4, 6 and 24 h).

The tail-flick assay is a pain receptive model which measures animal nociceptive response latencies to thermal stimulus, based mainly on spinal response [30]. It is known that the response to pain and the reaction time to painful stimulus are mediated at the central nervous system level, and more specific, at the spinal level [31]. It is consider that the substances which present an increase in reaction time to painful stimulus, compared to the control, are considered to have analgesic potential. In our study the influence of chemical modulation of ibuprofen on pain inhibition effect was noticed. It could be appreciate that the best influence was showed by radicals 4-NH_2_, 4-CN and 4-CH_3_, which substitute the aromatic ring of thiazolidine-4-one scaffold, the corresponding compounds (**4 m**, **4 k** and **4 h**) being more active than ibuprofen.

Writhing assay is a model used to evaluate the inhibition effect of visceral pain, considering the behavioral modifications, in terms of abdominal contortions [32]. The most intense peripheral analgesic effect was recorded for **4 h** (*R =* 4-CH_3_) and **4e** (*R =* 2-NO_2_), for which the analgesic effect was higher than of ibuprofen. The findings of analgesic assays based on central and visceral mediated pain, suggest that both mechanisms are involved in the anti-nociceptive activity of the tested compounds.

## Conclusions

In this study the toxicity degree as well as the anti-inflammatory and analgesic effects of new ibuprofen derivatives with thiazolidine-4-one scaffold, using in vitro and in vivo assays have been reported. The results highlighted the therapeutic potential of four ibuprofen derivatives (**4 m, 4 k, 4e, 4d**) for different disorders where inflammation and pain play an important role such as inflammatory, neurodegenerative and cancer diseases.

## Data Availability

The data supporting the conclusions of this article are incuded in the article. The supplementary can be requested from the corresponding author.

## Abbreviations

NSAIDs: Non-steroidal anti-inflammatory drugs
DMEM: Dulbecco’s Modified Eagle Medium
BFS: Bovine Fetal Serum
P/S/N: Penicillin/streptomycin/neomycin
MTT: 3-(4,5-dimethyl-2-thiazolyl)-2,5-diphenyl-2H-tetrazolium bromide
DMSO: Dimethylsulfoxide
SD: Standard deviation
LD_50_: Lethal dose 50
b.w.: Body weight
